# The Impact of Process Conditions on Surge Current Capability of 1.2 kV SiC JBS and MPS Diodes

**DOI:** 10.3390/ma14030663

**Published:** 2021-01-31

**Authors:** Hongyi Xu, Na Ren, Jiupeng Wu, Zhengyun Zhu, Qing Guo, Kuang Sheng

**Affiliations:** 1 College of Electrical Engineering, Zhejiang University, Hangzhou 310027, China; xuhongyi@zju.edu.cn (H.X.); wujiupeng@zju.edu.cn (J.W.); zhuzhengyun@zju.edu.cn (Z.Z.); guoqing@zju.edu.cn (Q.G.); shengk@zju.edu.cn (K.S.); 2 Hangzhou Global Scientific and Technological Innovation Center, Zhejiang University, Hangzhou 311200, China

**Keywords:** SiC, JBS, MPS, implantation, surge

## Abstract

This paper demonstrated the impact of process conditions on the surge current capability of 1.2 kV SiC junction barrier Schottky diode (JBS) and merged PiN Schottky diode (MPS). The influence of ohmic contact and defect density produced by implantation was studied in the simulation. The device fabricated with high temperature implantation had less defect density in the implant region compared with room temperature implantation, which contributed to higher hole injection in surge current mode and 20% surge capability improvement. In addition, with lower P+ ohmic contact resistance, the device had higher surge capability. When compared to device fabrication with a single Schottky metal layer in the device active area, adding additional P+ ohmic contact on top of the P+ regions in the device active area resulted in the pn junctions sharing a greater portion of surge current, and improved the devices’ surge capability by ~10%.

## 1. Introduction

Silicon carbide (SiC) diodes have shown great advantages in high temperature, high frequency, and high voltage applications [1]. Junction Barrier Schottky (JBS) diode with low reverse leakage current and low forward voltage drop is widely used in temperature sensors, high power systems, and radiation detection (alpha particle [2] and x/gamma radiation [3]) applications. A power system may develop system failure, and power diodes are required to endure a high surge current, otherwise a thermal runaway may result. Therefore, surge capability has been regarded as one of the most important reliability aspects of power diodes.

During the surge event, huge heat can be generated and dissipated within the device, thus leading to raised junction temperature and device failure. To improve the surge current capability of the JBS diode, the device voltage drop in surge current mode must be reduced to limit heat generation. The Merged PiN Schottky (MPS) diode was proposed based on the JBS structure with large P+ regions embedded in a SiC surface, beneficial in gaining PN junctions and a bipolar current conduction mode. During the surge event, the PN junction formed by the large P+ regions can be turned on and helps to reduce the device voltage drop significantly [4,5]. Since the first demonstration of the MPS diode [6], it has been shown that the P+ area percentage and cell design of the MPS structure influence the surge capability of the diode [7,8]. However, the device fabrication process technologies also have strong effects on the surge capability, especially the P+ region implantation and P+ ohmic contact process [9]. For the P+ region implantation, the implantation dose and temperature can affect the surface morphology and crystal quality of the implant region [10,11]. The surface and crystal defects can reduce the minority carrier density, and thus reduce the bipolar current. Therefore, the surge current capability of the MPS diode is weakened. For the P+ ohmic contact process, an additional metal layer is always added into the process flow of the MPS diode in the traditional fabrication method [12,13]. The additional metal process and the ohmic contact annealing process increase the complexity and fabrication cost. On the other hand, the device yield can be decreased. To address these problems, different implantation processes have been applied to the MPS diode fabrication, and the devices’ performance and surge current reliability compared. In addition, new device fabrication technologies have been developed to delete the P+ ohmic contact metal and annealing process without the sacrifice of performance and surge current capability. Devices were fabricated, and the experimental results demonstrated herein.

## 2. Materials and Methods

In this section, the simulation of the different structural parameters and process parameters are compared and analyzed. In order to confirm the mechanism, different process parameters were designed in the fabrication of silicon carbide JBS/MPS. The static characteristic of devices fabricated by different processes were measured and the surge capability of the devices compared.

### 2.1. Simulation Study of SiC MPS Diodes

#### 2.1.1. Different Contact Type Between the P+ Implant and Anode Metal

In order to relieve electric field crowding and prevent premature breakdown, a transition region consisting of a wide P+ implant is designed between the active region and termination region for a power device. The P+ implant of the transition region is connected to anode metal and the contact between the anode metal and the P+ implant can be processed as ohmic contact or Schottky contact.

**Figure 1:**
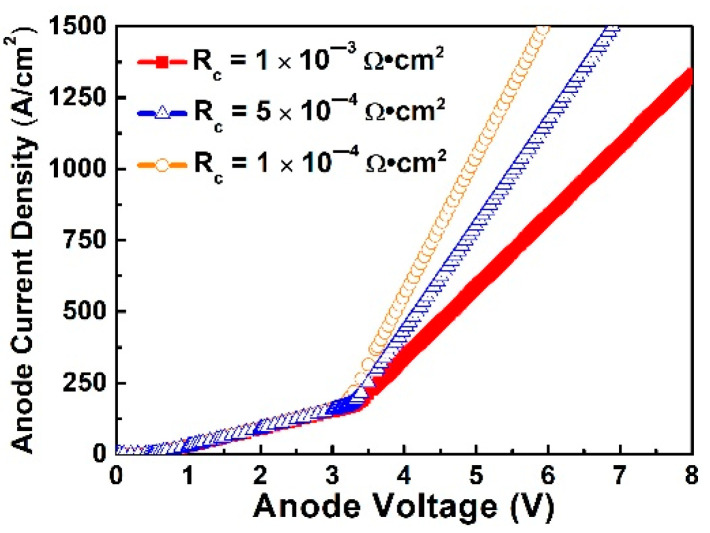
The turn-on behavior in the transition region with the varied ohmic contact resistance.

In normal conditions, the diodes work in unipolar mode. The PN junctions in the diode do not conduct current as the turn-on voltage of a SiC PN junction is as high as 2.7 V, thus P-ohmic contact does not contribute to device resistance in normal operation. However, when the surge current flows into the diode, the PN junction will be turned on and bipolar current injection will be triggered. In this case, the P-ohmic contact contributes to device resistance, and the lower contact resistance helps to decrease the heat generation in surge events. According to previous experimental results [14,15,16], the P-ohmic contact resistance can range from 1 × 10^−3^ Ω·cm^2^ to 1 × 10^−5^ Ω·cm^2^ which is affected by the P+ implantation and ohmic contact annealing process conditions. In the simulation, the P-ohmic contact resistance was set as 1 × 10^−3^ Ω·cm^2^, 5 × 10^−4^ Ω·cm^2^, and 1 × 10^−4^ Ω·cm^2^. The simulation results of the JV characteristics are shown in Figure 1. The results show that the diode behaves like a pure SBD diode when the anode voltage is below 3 V while the PN junction can be turned on at a higher voltage as shown from the rapidly increased JV slope. This means the diode enters bipolar mode. When comparing the three curves, it was found that the contact resistance not only affects the PN junction turn-on voltage but also the JV slope (i.e., device resistance in bipolar mode). In this paper, the PN junction turn-on voltage is defined as the anode voltage at a current density of 1384 A/cm^2^. The lower contact resistance results in lower resistance in the bipolar mode. Thus, lower P-ohmic contact resistance results in lower PN turn-on voltage and less power dissipation.

#### 2.1.2. Defect and Trap Density in P+ Region Caused by Implantation

**Figure 2:**
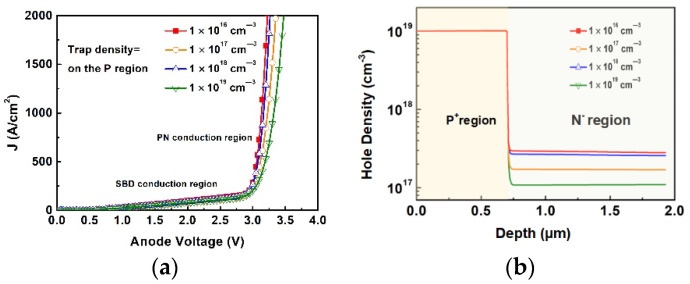
(**a**) The turn-on behavior in transition region with the varied trap density (**b**) hole density distribution in different trap density.

To gain good blocking performance, the P+ region is usually formed by implantation with a high Al^+^ dose. However, due to ion collision, the implanted ions cause damage in the lattice and produce defects that capture holes and reduce the number of holes injecting into the drift region during a high current conduction mode (for example a surge event). Defect density can be greatly affected by implantation dose, implantation energy, implantation temperature, and annealing temperature. In the simulation, different trap densities were assigned into the P+ region of MPS diodes, and the simulation results of forward IV characteristics are shown in Figure 2a. It was shown that with a higher trap density, the PN junction turn-on voltage (defined as the anode voltage at anode current density of 1384 A/cm^2^) is increased, and the slope of the JV curve is decreased accordingly. The minority carrier distribution is shown in Figure 2b, lower defect density would cause higher hole injection in the N region and result in lower resistance.

**Figure 3:**
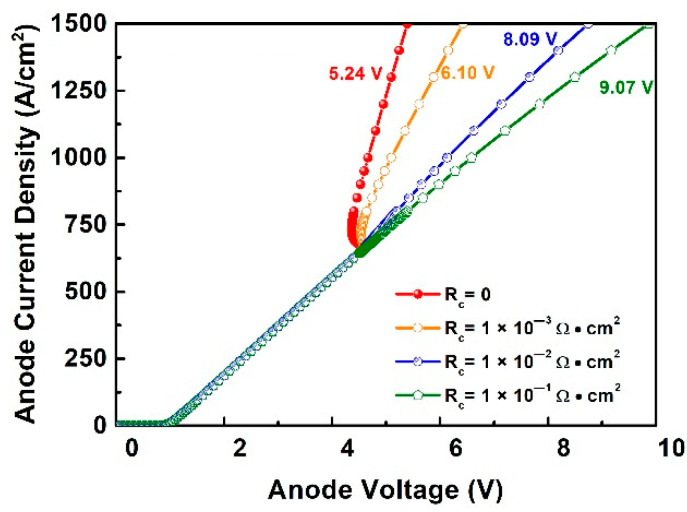
The turn-on behavior in the transition region with the varied ohmic contact resistance and high trap density.

The turn-on behavior is not only affected by the ohmic contact resistance but the defect density as well, the combination simulation of ohmic contact resistance is necessary. The different contact resistances 0.1 × 10^−3^ Ω·cm^2^, 1 × 10^−2^ Ω·cm^2^ and 1 × 10^−1^ Ω·cm^2^ with high trap density (1 × 10^18^ cm^−3^) were applied in the simulation. The results are shown in Figure 3. When the contact resistance is low, a snapback phenomenon is observed. And the lower contact resistance leads to lower PN turn-on voltage. The PN junction turn-on voltage at the anode current density of 1384 A/cm^2^, reduces from 9.07 V to 5.24 V when the ohmic contact resistance reduces.

### 2.2. Device Fabrication

**Figure 4:**
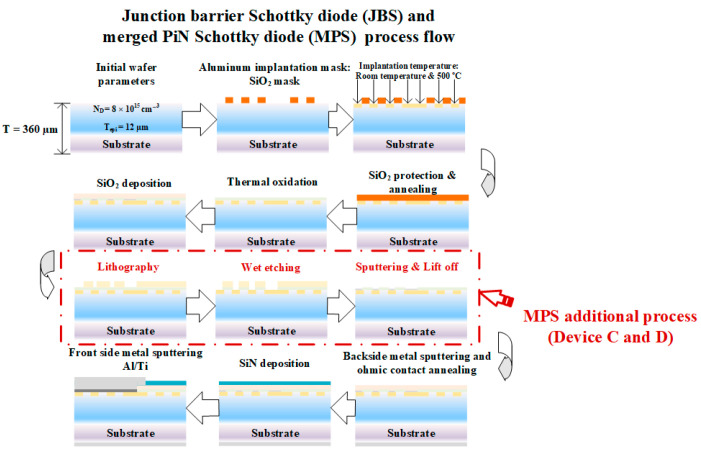
Junction barrier Schottky diode (JBS) and merged PiN Schottky diode (MPS) process flow.

**Figure 5:**
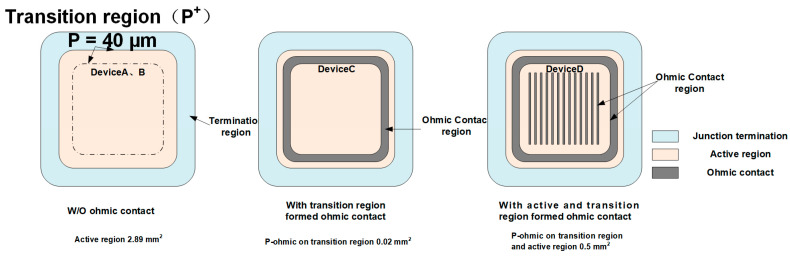
Three layout types of Device A, B, C, D. Device A and B are JBS, Device C and D are MPS.

The process flow of JBS and MPS is shown in Figure 4. Diodes were fabricated on n-type 4 off-axis epilayer 4H-SiC. The donor concentration is 8 × 10^15^ cm^−3^ and epilayer thickness 12 μm. The total thickness of the wafer is 360 μm and the resistivity of the substrate is 0.021 Ω·cm. In the process, the aluminum ions are implanted through the mask of SiO_2_ to form highly-doped P+ grids (>1 × 10^20^ cm^−3^ and the total dose is 2.12 × 10^15^ cm^−2^). Post-implantation annealing is operated at 1550 °C in the Argon atmosphere during which the wafer is protected with a shielding layer on the front side. Then, backside contact is formed by sputtering and annealing, followed by passivation patterning and frontside metal processing. Different process conditions like ion implantation temperature and ohmic contact geometry were studied in this work. Therefore, the devices were divided into four batches, two of them processed with different implantation temperatures: Room temperature (device A) and 500 °C (device B). By using hot implantation, the sheet resistance of the Al-implantation region under low anneal temperature (1550 °C) is lowered [5,17]. With hot implantation, the defect density is reduced, due to lattice repairing during the implantation process. Another two Devices (Device C and Device D) are MPS diodes with different ohmic contact geometry as shown in Figure 5. The process flow of the MPS diode needs one more photomask to form ohmic contact. The active area of the devices was the same, 2.89 mm^2^. The ohmic contact area in Device C was only 0.02 cm^2^ and Device D 0.5 cm^2^ with almost 25 times larger P-ohmic region by implementing the ohmic contact in the active region.

## 3. Results

### 3.1. Static Characteristic

#### 3.1.1. Device Fabricated by a Different Ion Implantation Temperature

**Figure 6:**
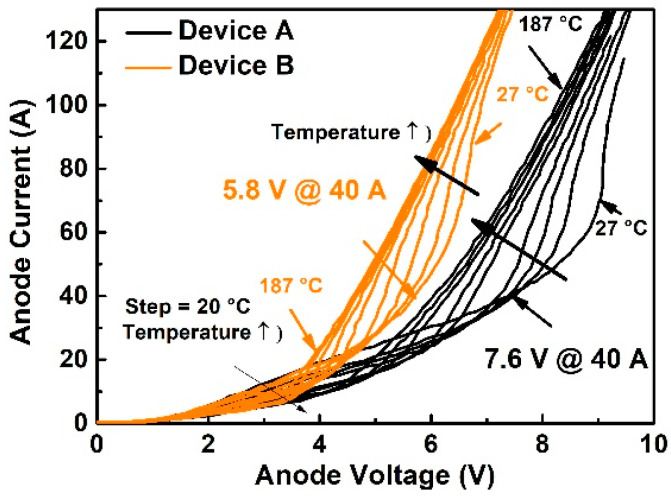
JBS forward characteristic at elevated temperature.

The forward IV characteristics of Device A and Device B measured at varied temperatures from 27 °C to 187 °C are shown in Figure 6, where the black lines are those for Device A and the orange lines are for Device B. The PN junction turn-on voltage for Device A is 8 V while that for Device B is only 5.4 V at an anode current of 40 A. If we calculate the power generation by multiplying the current with voltage, the power generation of Device B at a current level of 40 A is almost 30% lower than that of Device A. From the figure, the PN junction turn-on voltage decreases with the increasing temperature while the unipolar resistance (i.e., the slope of the IV curve before PN junction turning on) increases with it. The PN junction turn-on voltage of Device B decreases almost 0.8 V at 187 °C when compared to that in the room temperature case. The decrement of PN junction turn-on voltage of Device B is lower than Device A.

In addition, the IV curves in bipolar mode exhibit different slopes for different devices at different ambient temperatures, which means the device has a different bipolar resistance. Different differential resistances for Device A and B may be ascribed to the different minority carrier injection efficiency from the P+ implant region as they are processed with different implantation temperatures. According to the simulation results in Section 2.1.2, the various defect densities caused by the implantation process can lead to different minority carrier injection efficiency. High implantation temperature results in lower defect density in the implant region and higher minority carrier injection efficiency. Therefore, the bipolar resistance can be reduced when the implantation is performed at 500 °C (Device B).

#### 3.1.2. Comparison of JBS and MPS Diodes

To compare the contribution of the active region and the transition region to the surge current, the device C and device D with different ohmic contact geometries were designed and fabricated. The sketches of the two devices are illustrated in Figure 5. Device A is a JBS diode, Device C is an MPS diode with ohmic contact only formed in the transition region, and Device D is an MPS diode with ohmic contact formed in all of the P+ region.

**Figure 7:**
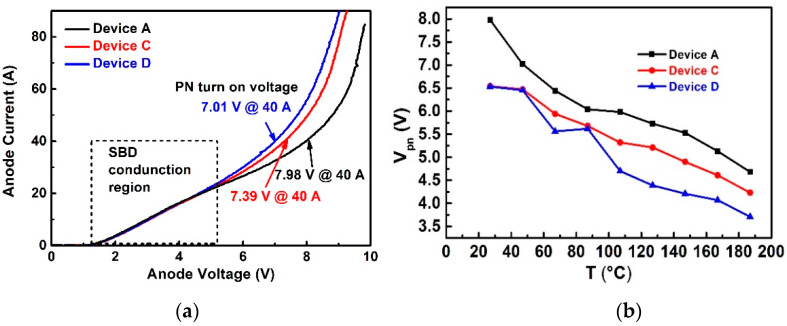
Comparison of the forward characteristics of devices A, C, and D (**a**) the forward characteristics of the device A, C, D at room temperature (**b**) The summary of the PN junction turn on voltage extracted from the IV characteristics measured at various temperatures ranging from 27 °C to 187 °C.

The experimental results of forward IV characteristics for Device A, C, and D are illustrated in Figure 7a. The SBD conduction region of the three devices is almost the same. The only difference among the three devices is PN junction turn-on voltage. With the 900 °C Ni-based ohmic contact annealing, the turn-on voltage of devices D is 1.5 V smaller than Device A, and 0.1 V smaller than Device C. The ohmic contact area in Device C is only 0.02 cm^2^ and Device D is 0.5 cm^2^ with almost 25 times larger P-ohmic region by implementing the ohmic contact in the active region. The larger P-region contributed to significant bipolar current under the high voltage. It is better to form ohmic contact (by applying the annealing process for the contact layer, the case for Device D) instead of Schottky contact (without annealing, the case for Device A) on top of the P+ region.

Additionally, the forward IV characteristics of the devices were measured at elevated temperature (ranges from 27 °C to 187 °C), the PN junction turn on voltages can be extracted from the IV curves and the results are summarized in Figure 7b. The PN junction turn-on voltages (V_pn_) of the three devices at room temperature are 7.98 V (Device A), 7.39 V (Device C), and 7.01 V (Device D), respectively. The PN junction turn-on voltage decreases with increasing temperature and the gap between Device C and D increases with elevated temperature. The reason is that the narrow PN junctions in the active region turn on at lower voltage at elevated temperature and the active region with P+ ohmic contact in Device D contributes a larger portion of the current and further lowers the device voltage drop.

#### 3.1.3. Analysis of the Damage Induced by the Different Implantation Processes

**Figure 8:**
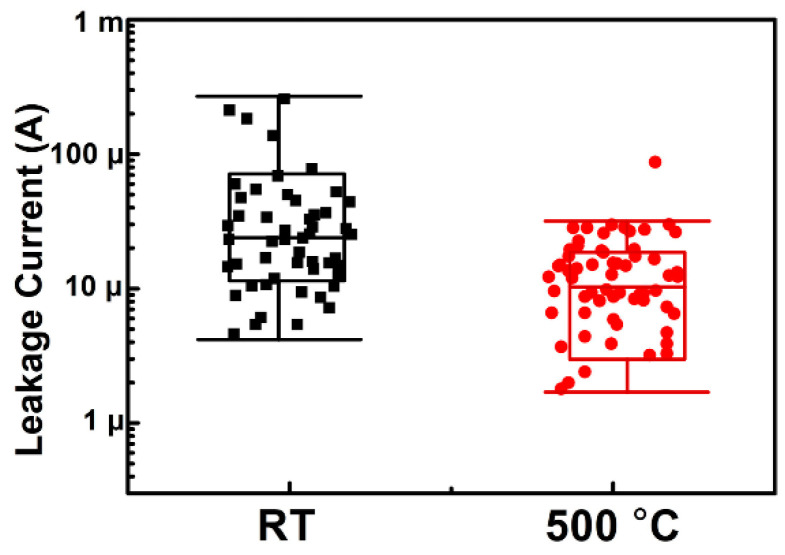
JBS block leakage current at 1200 V.

**Figure 9:**
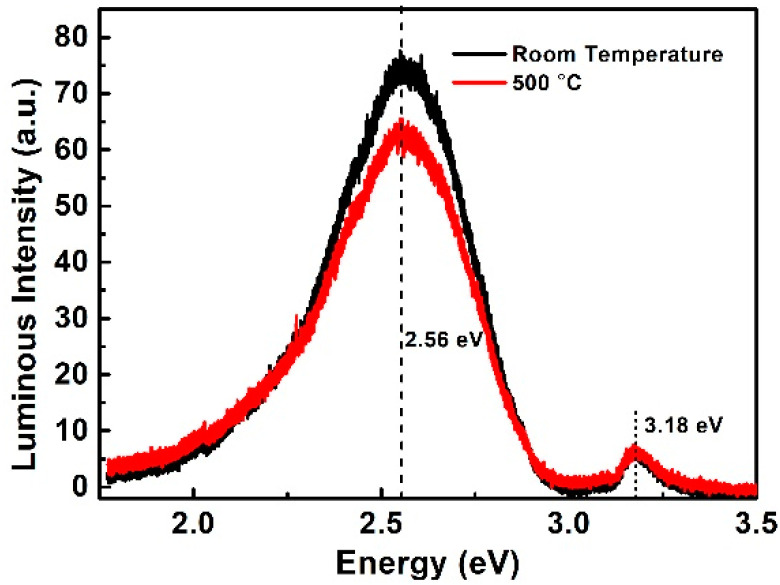
PL spectra of two samples implanted at room temperature and 500 °C.

The implantation area is vital in the JBS/MPS diode. During the implantation, ion collision causes a defect in the SiC and a portion of the defect recovers by the post-implantation. Under the blocking state, the bottom of the P+ region could be depleted, and the defect in the depletion could produce additional trap assistant leakage. On the SiC wafer fabricated by room temperature implantation, 50 pieces of devices are placed having the same design as Device A. While 50 pieces of device with the same design of Device B are also fabricated with 500 °C implantation. The leakage current at 1200 V of 50 pieces of devices was measured and analyzed. The analysis results are shown in Figure 8. The JBS fabricated by 500 °C implantation shows lower leakage than that fabricated by room temperature implantation (RT implantation) at the same voltage (1200 V). The lower leakage current represents a lower defect density of the implantation area. To measure the defect density, two wafers with different implantation temperature were prepared to conduct photoluminescence (PL) analysis with a 325 nm He-Cd excitation source. Before the post-implantation annealing, the crystal is seriously damaged. All of the photons are absorbed by the wafer and excited infrared light. In order to clarify the relationship between energy level (E) and luminous intensity, Figure 9 was plotted. The energy level was calculated by E = hc/λ. All luminous intensity data was one-tenth of the original data to make sure the maximum luminous intensity was lower than 100 a.u. The native peak was 3.18 eV near to the SiC bandgap energy and the defect energy level was 2.56 eV which is near to the literature value [18]. From the literature, if the maximum luminous intensity is normalized to 100 a.u. the luminous intensity of the high dose and low dose is 25 a.u. and 12.5 a.u. separately. The leakage of the channel region changes from 1 × 10^−8^ A to 1 × 10^−11^ A, which is due to the increment of trap-assistant leakage. In this experiment., except for the implantation temperature, Device A and Device B were fabricated by the same process. It is believed that the higher leakage of Device A is ascribed to the higher defect density induced by the room temperature.

#### 3.1.4. P+ Layer Sheet Resistance and Ohmic Contact Resistance

The factor of ohmic contact affected by the implantation process is surface roughness [19] and implantation activation rate [20]. The surface roughness after implantation is almost the same. The Van der Pau pattern is used to test the activation rate of the P+ region formed by room temperature and 500 °C implantation. The transmission line model (TLM) pattern was used to extract ohmic contact resistance in the three structures: (1) as-deposit aluminum/titanium on 500 °C Al implantation (2) as-deposit aluminum/titanium on room temperature Al implantation (3) Ni annealed at 900 °C on room temperature Al implantation.

**Figure 10:**
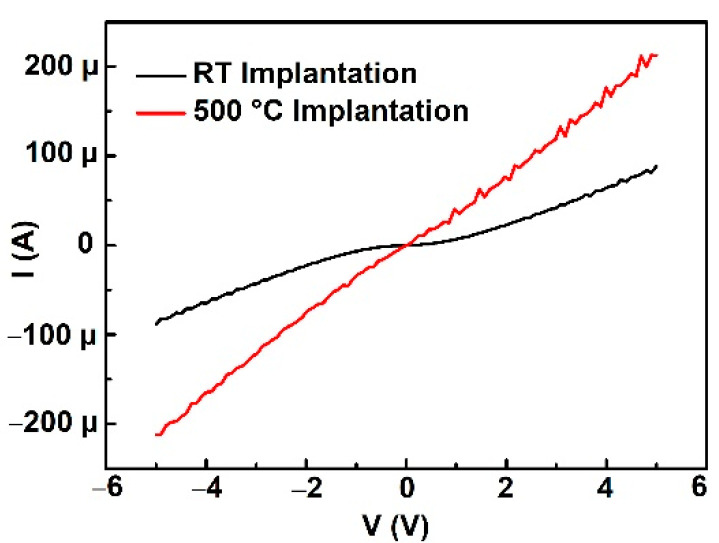
IV characteristics of TLM pattern structure on RT implantation sample and 500 °C implantation sample.

In the TLM test pattern, the as-deposited aluminum/titanium shows ohmic behavior with the help of 500 °C aluminum implantation, whereas it is a rectifying characteristic for the room temperature aluminum implantation sample as shown in Figure 10. The difference is mainly caused by different activation rates.

Through Hall measurement on the Van der Pauw test structure, the activated aluminum is only 5% of the total dose for the room temperature implantation sample, whereas it is 12% for the 500 °C implantation sample. If corrected data rH is used, considering the results in the literature [21,22,23], a different active ratio is obtained and the actual active ratio of the room temperature decreases from 5% to 2.5%. While the actual active ratio of the high temperature is decreased from 12% to 6%. A higher activation rate (3.5–7%) was extracted in the 500 °C implantation sample compared with room temperature implantation, even when considering the corrected rH.

**Figure 11:**
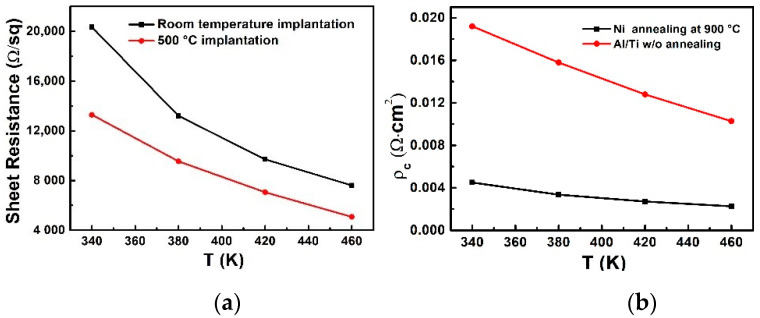
(**a**) The P-region sheet resistance tested at elevated temperature (**b**) the P ohmic contact resistance tested at elevated temperature.

The higher activation rate leads to higher active dopant concentration at the implant region surface. The native barrier gradually becomes narrower and results in more tunneling current through the metal to the semiconductor and thus ohmic contact is formed by as-deposited Al/Ti. Furthermore, during surge events, self-heating is significant in the device. The implantation sheet resistance and ohmic contact resistance were measured at elevated temperature and the results are shown in Figure 11. A higher activation rate of the holes leads to lower sheet resistance. Activation rate is determined by the density of the atom replaced by the aluminum ion while the activation rate also relies on the ionization rate. The ionization rate of Al atoms is increased at elevated temperature. Thus, the sheet resistance of 500 °C implantation sample decreases from 13,500 Ω/sq (measured at room temperature) to about 5000 Ω/sq (measured at 460 K). While the sheet resistance of the RT implantation sample decreases from 20,000 Ω/sq to 8000 Ω/sq. With a higher hole concentration at the raised temperature, the ohmic contact resistance would also decrease. The as-deposited aluminum/titanium reacts as ohmic characteristics and shows a decrement of ohmic contact resistance.

### 3.2. Surge Current Test Setup

**Figure 12:**
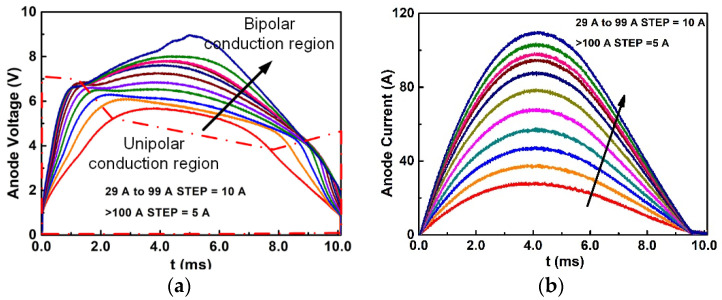

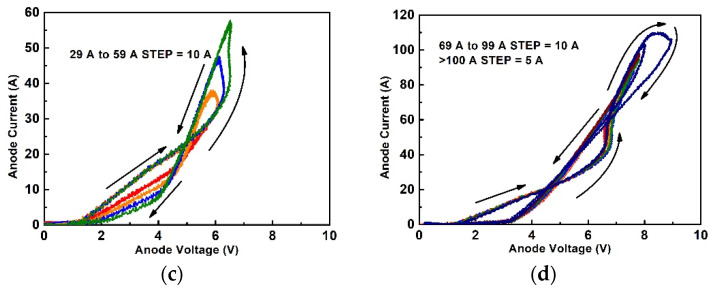
(**a**) Surge voltage waveforms of device B with the increased surge current. (**b**) Surge voltage waveforms of device B with the increased surge current. (**c**) Surge waveforms (30–60 A) of device B are replotted in the IV curve. (**d**) Surge waveforms (>60 A) of device B are replotted in the IV curve.

Device A and Device B were packaged in TO-220 and surge current tests were carried out to evaluate the surge current capability of the two types of device. The surge voltage waveform of Device B is illustrated in Figure 12a and the surge current waveform of Device B is illustrated in Figure 12b. The 10 ms half-sine wave surge current was applied to the device with a peak value gradually increasing from 30 A to 110 A. When the voltage is below the dashed-dotted line, the device is operated in unipolar conduction mode. When the voltage exceeds the line, the device enters bipolar mode, the hole formed by Al- implantation starts to inject into the drift layer causing a change of voltage slope. The replotted surge current (30–60 A)-surge voltage curve is illustrated in Figure 12c. The replotted current (>60 A)-voltage curve is illustrated in Figure 12d.

During the test, the peak of the surge voltage increases with the increment of the peak of the surge current. As shown in Figure 12a, when the peak of surge current is above 30 A, the surge voltage waveform is not sinusoidal. Two voltage turning points are shown in Figure 12a. The two voltage turning points are connected with the points in each surge voltage at a different surge current. The voltage turning point is caused by the conduction mode change. When the voltage is below the dashed-dotted line, the device is operated in unipolar conduction mode. When the voltage exceeds the line, the device enters bipolar mode, the hole formed by Al-implantation starts to inject into the drift layer and causes the change of voltage slope.

At 30 A, the replotted curve in Figure 12c, the second loop appears. In the second loop, the current increases slowly with first an increment of voltage. Then the current increases rapidly with little voltage increment, which means the PN junction starts to conduct the current leading to a decrement of the resistance. After this period, the voltage decreases with an increment of the current owing to the lowering PN band-offset caused by the elevated temperature. At the end of this period, the current reaches the maximum current. After the maximum current, heat generation becomes lower. The junction temperature starts to decrease and the current is decreased with the reducing voltage.

When the surge current was applicable to 60 A, the IV trajectories were replotted in Figure 12d. The IV trajectories gradually show the Third loop. In the third loop, due to the rapid temperature rise in this period, the resistance of the device increases quickly. This mechanism results in the negative resistance to positive resistance. Thermal destruction always occurs in this period.

## 4. Discussion

### 4.1. The Influence of Implantation Temperature on Surge Current Capability

**Figure 13:**
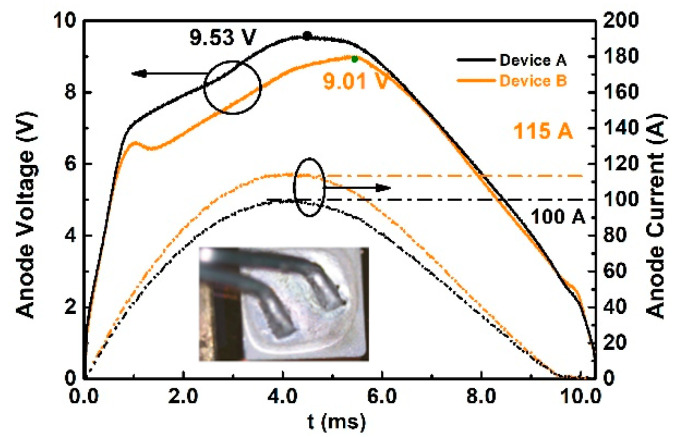
The voltage and current waveforms of Device A and B in the last test before failure.

**Figure 14:**
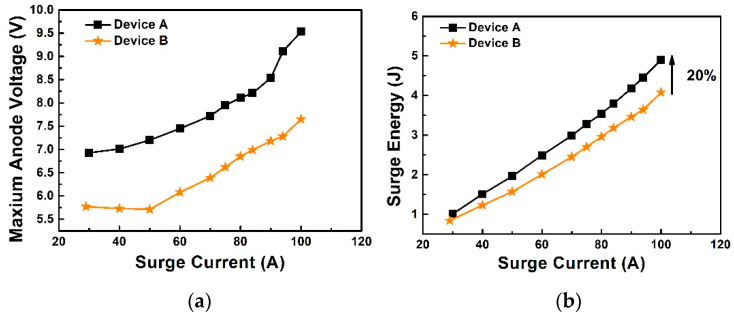
(**a**) Summary of maximum voltage at different surge currents for Device A and Device B. (**b**) Summary of dissipated energy at different surge currents for Device A and Device B.

After each surge test, the breakdown voltage is measured to check if the device has degraded. The last non-degradation waveforms are shown in Figure 13. Device A reaches 100 A at 25 °C, and its breakdown voltage is reduced from 1500 V to 900 V. Device B with the same measurement condition could reach 115 A with the breakdown voltage being reduced from 1550 V to 1000 V. The failure of the surge capability is over-temperature as shown in the inset of Figure 13, the front aluminum melts and the device breakdown voltage is affected. The maximum anode voltage is extracted from the waveform illustrated in Figure 14a. Device B shows a lower maximum voltage than Device A at each surge current, which is consistent with the static forward characteristics presented in Figure 6. Surge energy is also extracted and the results are shown in Figure 14b, the heat generation of Device A at 90 A is almost 20% higher than Device B. The surge capability of Device B is 23 times the normal current (5 A) while Device A is only 20 times. The promotion of the surge capability in Device B is caused by lower P+ ohmic contact resistance (analysis in Section 3.1.4) and higher minority carrier injection efficiency due to the higher activation rate and lower defect density with the process condition of high temperature implantation, as illustrated in Section 2.1.2. The lower ohmic contact and higher minority carrier injection efficiency lead to lower bipolar resistance and reduce heat generation.

### 4.2. The Influence of Ohmic Contact Area on Surge Current Capability

**Figure 15:**
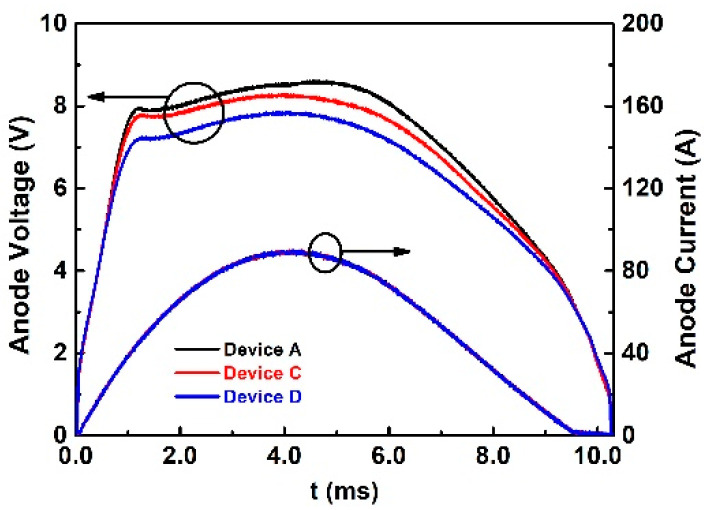
Surge waveforms of Device A, Device C, and Device D at surge current equal to 90 A.

The effects of ohmic contact geometry on the static IV characteristics and forward conduction performances of the diodes were illustrated in Section 3. Furthermore, the surge current capability of the diode can also be influenced by the different ohmic contact geometry design. In this sub-section, the surge current tests were performed on the three Devices, Device A, C, and D. The experimental results of surge current and voltage waveforms are shown in Figure 15. The three devices show different voltage waveforms when tested at the same peak surge current (90 A). The influence of ohmic contact resistance towards the I-V curve is exhibited in Section 2.1.1. With the help of large area ohmic contact on P-regions, Device D has the lowest voltage drop and better surge capability. The ohmic contact region is almost 25 times larger compared to Device C.

**Figure 16:**
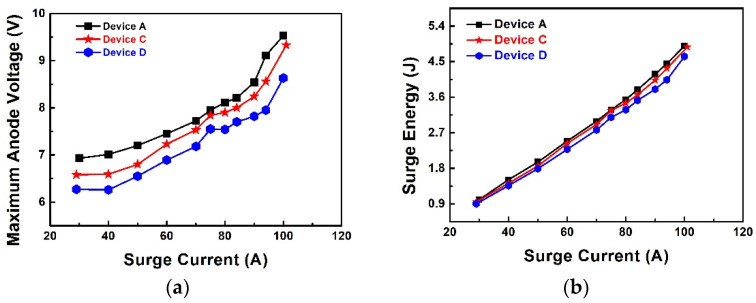
Summary of (**a**) maximum voltage and (**b**) dissipated energy at different surge current for Device A, Device C, and Device D.

The summary of maximum voltage and dissipated energy at different surge currents is illustrated in Figure 16. With an increasing surge current, the voltage difference between Device A and C decreases, while that for Device D is still 0.6 V less than that of Device C. In the low surge current range (30–50 A), due to the long P+ region in the transition region (width = 40 μm), the hole injection starts to inject in this period at the transition region. The ohmic contact on the large transition region (Device C and Device D) could lead to lower PN junction turn-on voltage and lead to a lower peak anode voltage. When compared with Device A at the same peak surge current (40 A), Device C has 0.5 V lower voltage, while Device D has 0.7 V lower voltage. In the high surge current range (>50 A), the maximum anode voltage of Device C becomes close to Device A. At peak surge current of 80 A, Device C has 0.2 V lower maximum voltage, while Device D has 0.7 V lower maximum voltage compared with Device A. Under high surge current, the P+ region (width = 3 μm) in the active region starts to endure a larger surge current in Device D due to lower ohmic contact resistance. Compared with Device A and Device D, Device C has a rapid maximum anode voltage increment during peak surge current at 50–80 A. At this period, the P+ in the active region starts to take a major part in conducting the surge current and gradually generates the main parts of the heat. Due to the Schottky contact on the active region (Device C and Device D), the surge energy of these devices is higher than Device A.

The surge energy was calculated, the device D with ohmic contact produce 3.8 J, while the device C and device A produce 4.03 J and 4.18 J separately at a surge peak current of 90 A. The device D has ~10% less heat generation than Device A and ~6% less heat generation than Device C at this surge current. Assuming the devices are destroyed at the same energy (e.g., 4 J), Device D is destroyed at 100 A, while Device A and Device C are destroyed at 90 A and 92 A separately.

## 5. Conclusions

In this paper, the influences of different process conditions such as ion implantation temperatures and ohmic contact geometries on the device performance and surge current capability were studied for the 4H-SiC JBS and MPS diodes. Based on the simulation and experimental results, the high temperature implantation could lower the contact resistance on the P+ region and reduce the defect density in the implant region. The lower contact resistance can reduce the PN junction turn-on voltage and the bipolar resistance. Device A fabricated with high temperature implantation had less defect density in the implant region compared with room temperature implantation, which contributes to higher hole injection in surge current mode and 20% surge capability improvement. In addition, with lower P+ ohmic contact resistance, the device had higher surge capability compared with Device A and Device C. When compared to Device C fabricated with single Schottky metal layer in the device active area, adding additional P+ ohmic contact on top of the P+ regions in the active area (the case for Device D) made the PN junctions share a greater portion of high surge current, and improve the devices’ surge capability by ~10%. Similarly, surge capability was observed at low surge current, due to the large transition region with P-ohmic.

## Data Availability

Data is contained within the article.
